# Losing track of time through delayed body representations

**DOI:** 10.3389/fpsyg.2015.00405

**Published:** 2015-04-13

**Authors:** Thomas H. Fritz, Agnes Steixner, Joachim Boettger, Arno Villringer

**Affiliations:** ^1^Max Planck Institute for Human Cognitive and Brain SciencesLeipzig, Germany; ^2^Department of Nuclear Medicine, University of LeipzigLeipzig, Germany; ^3^Institute for Psychoacoustics and Electronic MusicGhent, Belgium

**Keywords:** time perception, body schema, visual delay, virtual mirror, flow, intoxication

## Abstract

The ability to keep track of time is perceived as crucial in most human societies. However, to lose track of time may also serve an important social role, associated with recreational purpose. To this end a number of social technologies are employed, some of which may relate to a manipulation of time perception through a modulation of body representation. Here, we investigated an influence of real-time or delayed videos of own-body representations on time perception in an experimental setup with virtual mirrors. Seventy participants were asked to either stay in the installation until they thought that a defined time (90 s) had passed, or they were encouraged to stay in the installation as long as they wanted and after exiting were asked to estimate the duration of their stay. Results show that a modulation of body representation by time-delayed representations of the mirror-video displays influenced time perception. Furthermore, these time-delayed conditions were associated with a greater sense of arousal and intoxication. We suggest that feeding in references to the immediate past into working memory could be the underlying mental mechanism mediating the observed modulation of time perception. We argue that such an influence on time perception would probably not only be achieved visually, but might also work with acoustic references to the immediate past (e.g., with music).

## Introduction

Body representation and sense of time in humans seem to be closely related. Previous [1.4cm]4 research showed that several physiological parameters such as heart rate (Wittmann, 2013) and body temperature (Wearden and Penton-Voak, 1995) modulate the way we perceive time. Also, the mental representation of one’s own body, the body schema, seems to play an influential role in the subjective perception of time. Body schema has been defined as the “mental construct that comprises the sense impressions, perceptions, and ideas about the dynamic organization of one’s own body and its relations to that of other bodies” (Berlucchi and Aglioti, 1997). The perception of body schema can be modified by a variety of means (Holmes and Spence, 2006). An established method to achieve this is in an experimental setting where visual feedback is presented via mirrors or video monitors (Holmes and Spence, 2004; Bernardi et al., 2013).

Such manipulations of body schema can be employed to create proprioceptive/sensory changes and illusions such as, for example, the rubber hand illusion (Holmes and Spence, 2007), and out-of-body experiences (Lenggenhager et al., 2007). A manipulation of body schema, however, also seems to relate to recreational experiences that are associated with an altered perception of time. For example, the consumption of cannabis has been shown to affect both bodily representation and sense of time (Edelmann and Terbuyken, 2002; Atakan et al., 2012). Methods not related to intoxication, such as meditating, that employ changes in the monitoring of body schema have also been shown to have—among other psychological effects—an effect on the perception of time (Berkovich-Ohana et al., 2013). Likewise, the subjective experience of flow (a psychological state associated with high motivation and either motor or cognitive challenge) that often corresponds to a change in bodily experience appears to be associated with a modified perception of time (cf. Jackson and Csikszentmihaly, 1999).

Further evidence for a relation of body schema and time perception derives from schizophrenia research. Schizophrenia patients, who have been found to display a different body representation schema (Thakkar et al., 2011), showed greater variability with regard to their performance in a timing task and impaired ability to estimate time (Peterburs et al., 2013; Bolbecker et al., 2014).

Taken together, previous findings strongly suggest an association of one’s own body representation and one’s perception of time, so that a modulation of own body representation might affect the ability to accurately estimate time. In order to further investigate this relationship, we designed an experiment in which we manipulated bodily representations via delayed and non-delayed virtual mirrors. Because variables such as sex and age have been shown in the past to be associated with the perception of time, they were assessed to identify possible influences as well as moderator or mediator effects (Perbal et al., 2002; Glicksohn and Hadad, 2012).

## Materials and Methods

### Participants

Seventy individuals (39 females, age 30.01 ± 14.21, range: 11–67) took part in the experiment. Required minimal age to take part in the study was 10 years. Participants gave their written consent and did not receive any compensation.

### Procedure

**FIGURE 1:**
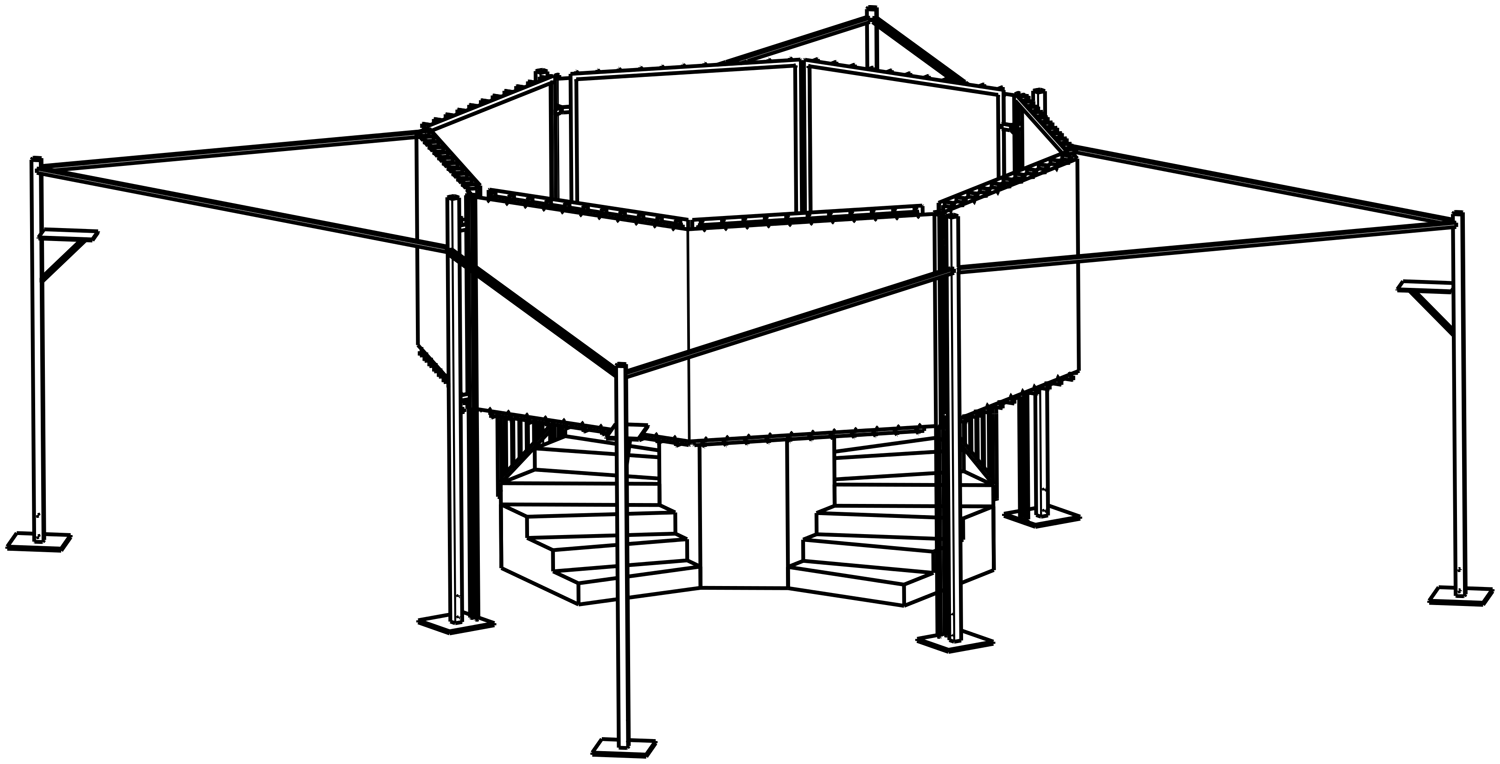
**Experimental installation “Kaleidoscope of Time.”** The participant enters the installation through a small staircase and stands on a tower, where eight video screens are visible that show video recordings/representations of him/her. Beamers projecting the video signals on back projection screens were positioned on the four columns in the periphery of the installation.

Each participant was randomly assigned to one of four groups. There were two experimental groups (aware delayed and non-aware delayed) and two control groups (aware not delayed and non-aware not delayed). We used a 2x2 design with delayed videos vs. non-delayed videos of body representations as one factor, and time-awareness vs. no time-awareness ^1^ as the other. In both the delay conditions, participants were presented with visual delayed videos of themselves, while in the two non-delayed groups videos were shown in real-time. Individuals were additionally assigned to one of the two “time-awareness” conditions (aware delay: *n* = 19; aware no delay: *n* = 20). In the awareness conditions they were instructed to enter the installation and return after they assumed 90 s to be over. Participants in the “no time-awareness” conditions (not aware delay: *n* = 16; not aware no delay: *n* = 15) were asked to enter the installation and stay there as long as they wanted. Prior to the experiment participants were instructed to fill out a questionnaire that also included constructs such as Situational Self-Awareness, Attentional Resource Allocation, Time Perspective, and Impulsiveness. Because the current paper aims to focus on a relation of body representation and time perception, results of these measurements are not reported here. Afterward, participants were asked to leave their timekeepers (i.e., cell phones, watches) with the experimenter for the duration of their stay in the experimental installation (**Figure 1**), allegedly in order to avoid distraction.

How long each participant stayed in the installation was unobtrusively measured by the experimenter. All participants received a post-experimental questionnaire including measures of arousal, perceived intoxication, subjective experience of changes in time, and several other scales not relevant to the current study, as well as some demographic questions.

A video signal was recorded by a camera installed above one of the eight video screens. This signal was then transferred to a media-server computer. A software (Wings Vioso, Duesseldorf, Germany) allowed to systematically delay the video signal and output eight different delayed versions (1–4 s) of the video signal during the delay condition. Groups of two outputs were transmitted to one of four beamers that projected the video signals on back projection screens (one on each of two screens), so that the participants could see the eight different visual presentations on all eight screens without artifacts (e.g., shadows).

### Data Analysis

Estimation error was computed in the awareness conditions as the measured duration of stay in the installation minus 90 s and in the no awareness conditions as the measured duration of stay minus the estimate of duration of stay by participants. Similar to previous research on human time estimation or production, we used absolute estimation error as an indicator of magnitude of estimation error, which is irrespective of its direction (Pastor et al., 1992; Rammsayer, 1997; Barkley et al., 2001a, b; Kerns et al., 2001). Further, following the procedures of Bauermeister et al. (2005), we applied logarithmic transformation on absolute estimation errors, i.e., estimation error plus 1 was computed and log-transformed, to reduce skewness of our data and to bring the raw absolute values more in line with the assumptions of parametric statistics. Because it was not possible to directly investigate differences between signed estimation errors due to properties of data distribution, we computed transformed signed estimation errors by adding the highest negative estimation error (195 s) to get positive values. In order to reach a normal distribution these values were squared afterwards. In order to compute effects on the direction of estimation errors, estimation errors greater than 0 s were coded as an overestimation of duration, and an error below 0 s was coded as an underestimation of duration.

The variables age and duration of stay were split into two sections by their median, i.e., younger vs. older age and long vs. short duration of stay. The cut-off for high vs. low arousal was defined as the value of three on the administered 6-point scale. Parametric computations (i.e., ANOVA, independent samples *t*-test) were used when requirements were met by the data. If basic assumptions were violated, non-parametric computations (i.e., Kruskal–Wallis test, Wilcoxon Signed-Rank test, *U*-test) were employed.

### Measures

#### Arousal

Experienced arousal was measured by means of the one-item Felt Arousal Scale (FAS; Svebak and Murgatroyd, 1985). Participants had to indicate their arousal on a 6-point scale with 1 indicating “low arousal” and six indicating “high arousal.” This measure of arousal or activation was found to correlate with other arousal scales ranging from 0.45 to 0.70, and thus showed convergent validity (Sheppard and Parfitt, 2008).

#### Intoxication

Participants indicated their perception of intoxication assessed by the question “Wie ‘berauscht’ fühlen Sie sich im Moment?” (“How ‘intoxicated’ do you feel at the moment?”) on a 4-point Likert scale with 1 indicating “not at all” and four indicating “very much.”

#### Subjective Changes in Sense of Time

Subjectively experienced changes in sense of time were assessed by the subdimension of “time sense” of the Phenomenology of Consciousness Inventory (PCI; Pekala et al., 1986). The overall measure was shown to have sufficient reliability and validity for assessing changes in phenomenological experience (Pekala et al., 1986). The subdimension used in this study consists of three items, each comprising two opposing statements. Participants rated the statements on a 5-point scale, indicating with which of the two statements they agreed more, e.g., a rating of three indicating equal agreement with both statements. Sample item: “Time seemed to greatly speed up or slow down” vs. “Time was experienced with no changes in its rate of passage.” In our study, Cronbach’s alpha was α = 0.83.

#### Other Outcomes and Controls

The experiences of feeling detached from one’s body and of “sinking into pictures” were assessed by single items constructed for the purpose of this study. These items were rated on a 4-point Likert scale ranging from “not at all” to “completely.” For the assessment of subjectively experienced waste of time the item “In your honest opinion, do you think this survey was a waste of time?” was used (O’Brien et al., 2011). In addition, participants in the time-awareness conditions were asked if they used counting to estimate time correctly. Finally, data on age and sex were collected.

## Results

We constructed a general linear model (GLM), including the variables *group*, *duration of stay* (long vs. short), *age* (younger vs. older) and *sex.* In addition, we modeled interactions between *sex* ×*group*, *age* ×*group,* and *duration of stay* × *group*. *Group* was found to have a significant effect on log-transformed estimation error, *F*(3,53) = 3.36, *p* = 0.025, η^2^ = 0.160. Moreover, a significant interaction existed between duration of stay and group, *F*(3,53) = 4.67, *p* = 0.006, η^2^ = 0.209. No significant main effects were observed for sex, *F*(1,53) = 3.13, *p* = 0.083, η^2^ = 0.056, duration of stay, *F*(1,53) = 0.08, *p* = 0.781, and age, *F*(1,53) = 0.05, *p* = 0.831. Further no significant interactions existed between sex and group, *F*(3,53) = 1.20, *p* = 0.319 or age and group, *F*(3,53) = 0.83, *p* = 0.485. The overall corrected model accounted for 42.8% of variance, *F*(15,53) = 2.65, *p* = 0.005 (for mean and standard deviation see **Table 1**).

**Table 1:** **Mean and standard deviation of absolute time estimation errors (log-transformed and non-transformed) for experimental and control groups**.

Experimental group	Log-transformed absolute estimation error			Absolute estimation error in s		
	
	Duration of stay					
	Short	Long	Total	Short	Long	Total
Aware delay	1.41 (0.23)	0.95 (0.51)	1.24 (0.42)	28.50 (16.03)	13.29 (14.40)	22.89 (16.82)
Aware no delay	1.49 (0.24)	1.34 (0.46)	1.42 (0.35)	33.64 (15.97)	34.33 (35.91)	33.95 (26.03)
Not aware delay	1.31 (0.37)	1.85 (0.39)	1.61 (0.46)	24.71 (15.86)	90.00 (56.30)	61.44 (53.95)
Not aware no delay	1.29 (0.28)	1.50 (0.31)	1.38 (0.30)	23.00 (18.59)	37.83 (29.93)	29.36 (24.26)
Overall	1.39 (0.27)	1.43 (0.52)	1.41 (0.40)	28.13 (16.38)	46.59 (47.59)	36.35 (35.06)

**FIGURE 2:**
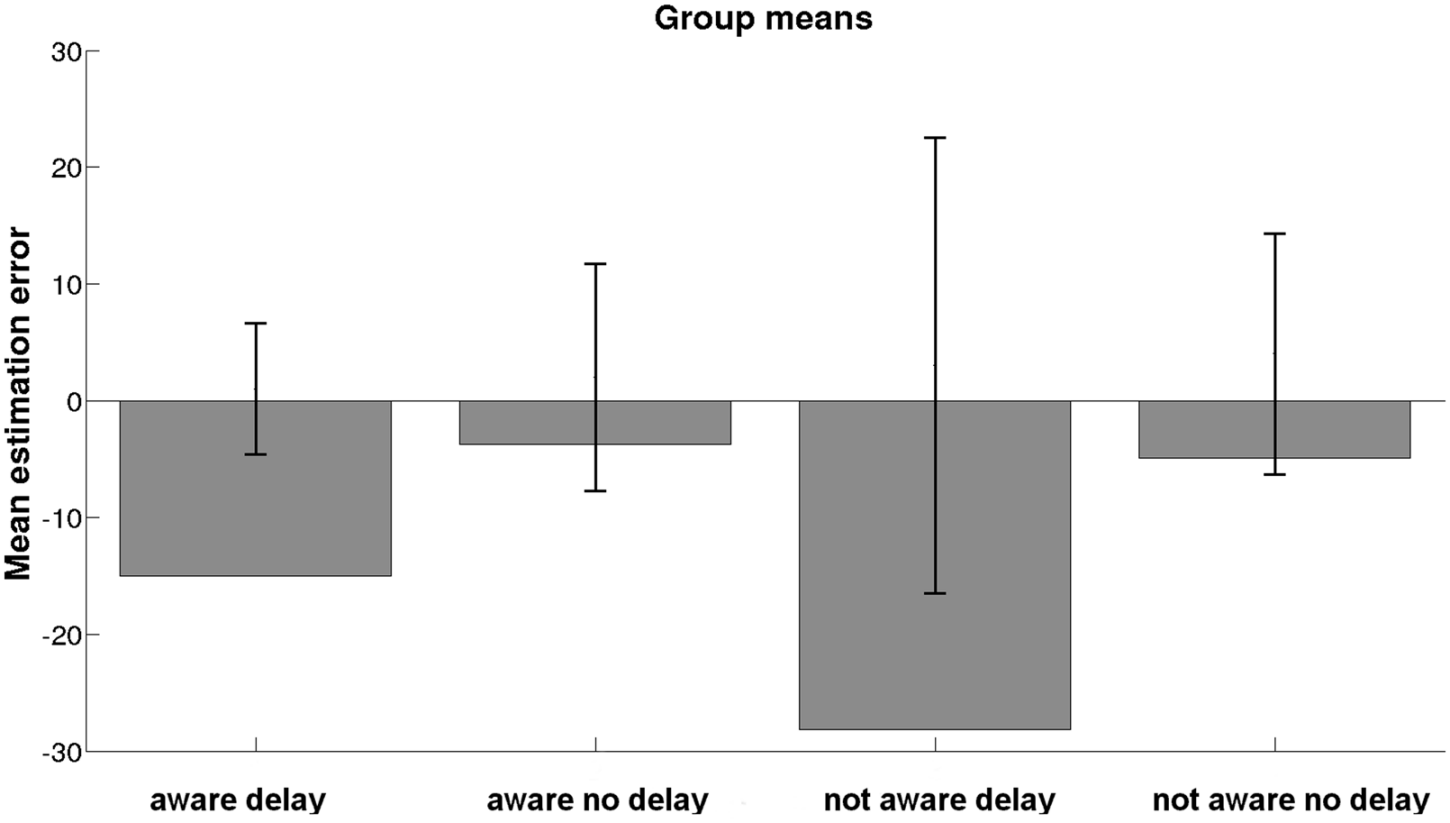
**Mean estimation errors for each experimental group; error bars show standard deviation**.

Due to the non-normality of data, *W*(69) = 0.945, *p* = 0.005, and inhomogeneity of variances, no ANOVA or Kruskal–Wallis test could be performed to detect possible effects of group on means of estimation error. A Welch test computed on the basis of the transformed signed estimation error did not yield any significant differences between groups, *W*(3,32) = 0.690, *p* = 0.565. However, the descriptive pattern of group means of estimation error is shown in **Figure 2**. The overall mean of estimation error was *M* = -12.75, SD = 49.04. A Kruskal–Wallis test did not yield any differences on the duration of stay between groups, χ^2^ = 1.880, *p* = 0.598.

No significant effect was found of groups on the direction of estimation error, i.e., overestimation vs. underestimation, χ^2^(3, *n* = 69) = 6.433, *p* = 0.092 ^2^. Furthermore, a Chi-Square test revealed neither significant differences between gender and direction of estimation error, χ^2^(1, *n* = 69) = 0.122, *p* = 0.727, nor between age groups and direction of estimation error (age < 25 vs. age > 24), χ^2^(1, *n* = 69) = 0.349, *p* = 0.555. Additionally, an ANOVA yielded no effect of perceived waste of time (three groups) by subjects on log-transformed absolute estimation error, *F*(2,66) = 0.454, *p* = 0.637.

With regard to subjective changes in the perception of time, we found that groups differed significantly on the degree of perceived changes in time sense, χ^2^(3, *n* = 70) = 8.674, *p* = 0.034. According to ranks, participants of the *aware delayed group* experienced the strongest subjective changes in the passage of time, followed by the *aware no delay* and *not aware delay* groups. Participants in the *not aware no delay* group reported the lowest changes in time sense.

Further, a Mann–Whitney *U*-test showed a significant effect of delay condition (delay or no delay) on the feeling of arousal, *U* = 387,500, *p* = 0.009 ^3^, on perceived intoxication, *U* = 338.500, *p* = 0.013 ^4^, and a marginal effect on the experience of ‘sinking into the pictures,’ *U* = 444.500, *p* = 0.038 ^5^. It did not have an effect on feeling detached from one’s body, *U* = 468.000, *p* = 0.071. With regard to the experimental ‘awareness’ condition (when participants were instructed to stay in the installation for 90 s), no significant effects on arousal, *U* = 492.000, *p* = 0.219, perceived intoxication, *U* = 483.000, *p* = 0.873, ‘sinking into pictures,’ *U* = 596.000, *p* = 0.916, feeling detached from one’s body, *U* = 495.500, *p* = 0.171 ^6^, were found. A Kruskal–Wallis test did not yield any differences on the duration of stay between groups, χ^2^(3, *n* = 70) = 1.880, *p* = 0.598.

## Discussion

Here, we demonstrate an influence of temporally manipulated visual own-body representations on time perception and a number of other psychological parameters. A delayed video presentation of one’s own body image resulted in a distorted absolute time percept so that participants perceived durations of staying in the experimental setup either as much longer or much shorter than in reality. This shows that a manipulation of body schema by visual delay strongly corresponds to a modulation of time percept, which was observed regardless of whether participants had been instructed in the experimental paradigm to stay a certain amount of time (90 s), or to stay as long as they wanted. Such an association between changes in body schema and time perception has previously been reported, for example in schizophrenic patients who display a “natural” deviation of body schema compared to healthy controls (Thakkar et al., 2011). Furthermore, time perception is affected in individuals whose body schema was modulated by means of meditation (Berkovich-Ohana et al., 2013). Time perception is also different after the consumption of drugs, which often also leads to changes in body schema (Edelmann and Terbuyken, 2002; Atakan et al., 2012). The results of the current study are thus in accord with previous concepts postulating an influence of body schema on time perception, but it is the first time that visual delay of one’s own body has been used to manipulate time perception.

Note that while in the current study we manipulated the visual representation of the own body (thus an aspect of body schema), we more specifically also presented several visual representations of the own body at different time points simultaneously. This specific manipulation of body schema was thus also associated with feeding in references of the immediate past into working memory that is known to be crucial to time perception of durations in a seconds to minutes range (Buhusi and Meck, 2005; Allman et al., 2014). We argue that this has a parallel in the acoustic domain, where references to the immediate past through auditory-motor associations and operant conditioning play a major role in musical improvisations. Here a density of referential stimulation—different to the current visual experiment—would not be mediated by simultaneity (of several video signals), but by the mostly repetitive organization of music. Note, however, that no working memory related information was being ‘actively’ manipulated in the task, i.e., participants were for example not required to remember how their body position was a few seconds ago, so the proposed link to working memory discussed here has yet to be considered rather weak.

Furthermore, the manipulation of own-body representations led to a number of other psychological outcomes that had previously been evoked with musical interventions (Rickard, 2004) or by drug administration (Parrott et al., 2007). Participants who were presented with their delayed body representations reported significantly higher perceived intoxication and arousal. These effects were (similarly to the observed effects on time perception) independent from participants being instructed to stay a certain amount of time (90 s), or stay as long as they wanted.

Additionally, the current study replicated the findings of previous research on human timing in the seconds to minutes range, which showed a general tendency to overestimate time in the seconds to minutes range (Wittmann et al., 2010). This tendency was found in all experimental conditions in the current experiment.

A limit of the current investigation is that effects of the time delays utilized are not systematically investigated. We just compared delay on/off. For future studies it would, for example, be interesting to identify which time delays work best for corrupting the perception of time. Further, we did not investigate if a general effect of non-delayed body representations compared to no visual feedback of one’s body exists, another question that might be addressed in future studies. Previous research showed that attention can modulate perception of time (Brown, 1985). Accordingly, it is possible that attentional processes masked or contributed to effects by modulation of body schema on time perception, as we did not control for attentional load in this study. However, it seems rather unlikely that attention differed substantially between delay vs. non-delay groups because besides effects of group on time estimates, we also found effects of delay condition on outcomes completely unrelated to attentional processes, such as arousal or perceived intoxication. Previous research also reported a greater variability in time estimation tasks in individuals with an altered body schema, i.e., schizophrenic patients (Bolbecker et al., 2014). Due to the fact that we did only employ an in-between study design, we could not test for this directly in the current study. Nonetheless, descriptive differences in standard deviation between experimental groups may point to an effect of manipulation of body schema on time estimation performance variability. It would be interesting to focus more on this correlation of time perception and body schema in future work. In addition, it would be interesting to test the effects of delayed video representations over different durations in future studies, given that in the current study we only tested target durations of 90 s in the time awareness conditions.

In conclusion, we here report a novel method to modify the perception of time by modifying body schema through delayed video representations of the own body. In addition to effects on time perception, we furthermore report effects on perceived arousal and intoxication. We discuss possible parallels to the functioning of music in the acoustic domain with respect to presenting references to the immediate past.

## Conflict of Interest Statement

The authors declare that the research was conducted in the absence of any commercial or financial relationships that could be construed as a potential conflict of interest.

## Footnotes

1 Note that “time awareness” should be understood as relative to the non-awareness conditions, i.e., pointing to increased attention dedicated to time by participants compared to participants assigned to the no time-awareness conditions. This is due to the fact that we did not employ a control task that made sure that participants were constantly focusing on the passage of time in the time-awareness conditions.

2 Bonferroni–Holm corrected alpha level: α’ = 0.0167.

3 Bonferroni–Holm corrected alpha level: α’ = 0.0125.

4 Bonferroni–Holm corrected alpha level: α’ = 0.0167.

5 Bonferroni–Holm corrected alpha level: α’ = 0.025.

6 Bonferroni–Holm corrected alpha level: α’ = 0.0125.
